# The Year of Care approach: developing a model and delivery programme for care and support planning in long term conditions within general practice

**DOI:** 10.1186/s12875-019-1042-4

**Published:** 2019-11-08

**Authors:** Sue Roberts, Simon Eaton, Tracy Finch, Nick Lewis-Barned, Monique Lhussier, Lindsay Oliver, Tim Rapley, Dawn Temple-Scott

**Affiliations:** 1 Year of Care Partnerships, Northumbria Healthcare NHS Foundation Trust, Wansbeck Hospital, Ashington, Northumberland NE63 9JJ England; 2 Northumbria University, Coach Lane Campus, Benton, Newcastle upon Tyne, NE7 7XA England

**Keywords:** Long term conditions, Care planning, House of care, Self-management support, Practice development, Year of care, Implementation science

## Abstract

**Background:**

People with long term conditions (LTCs) make most of the daily decisions and carry out the activities which affect their health and quality of life. Only a fraction of each contact with a health care professional (HCP) is spent supporting this.

This paper describes how care and support planning (CSP) and an implementation framework to redesign services, were developed to address this in UK general practice. Focussed on what is important to each individual, CSP brings together traditional clinical issues and the person’s lived experience in a solution focussed, forward looking conversation with an emphasis on ‘people not diseases’.

**Methods:**

The components of CSP were developed in three health communities using diabetes as an exemplar. This model was extended and refined for other single conditions and multimorbidity across 40 sites and two nations, over 15 years. Working with local teams and communities the authors used theoretical models of care, implementation and spread, developing and tailoring training, support and resources to embed CSP as usual care, sharing learning across a community of practice.

**Results:**

The purpose, content, process, developmental hurdles and impact of this CSP model are described, alongside an implementation strategy. There is now a robust, reproducible five step model; preparation, conversation, recording, actions and review. Uniquely, preparation, involving information sharing with time for reflection, enables an uncluttered conversation with a professional focussed on what is important to each person. The components of the Year of Care House act as a checklist for implementation, a metaphor for their interdependence and a flexible framework. Spreading CSP involved developing exemplar practices and building capacity across local health communities. These reported improved patient experience, practitioner job satisfaction, health behaviours and outcomes, teamwork, practice organisation, resource use, and links with wider community activities.

**Conclusions:**

Tested in multiple settings, CSP is a reproducible and practical model of planned care applicable to all LTCs, with the capacity to be transformative for people with LTCs and health care professionals. It recaptures relational dimensions of care with transactional elements in the background. Options for applying this model and implementation framework at scale now need to be explored.

## Background

Long term conditions (LTCs) are the fastest growing cause of ill health and death worldwide. In the UK people living with LTCs use 60% of GP time and 70% of NHS resources [1]. Importantly, of these 40% have multiple conditions, most commonly in disadvantaged communities [2]. Caused by a complex mixture of genetics, culture, longevity and environmental issues LTCs cannot be cured but can be managed by a combination of traditional treatments and lifestyle and environmental changes. People living with LTCs make most of the decisions and adjustments that affect their health and quality of life. They may have to manage complex treatment regimens [3], and make substantial changes to their daily lives which have an impact on family and friends as well as their physical, emotional and economic resources [4]. Each person spends only a few hours each year with health care professionals (HCPs) discussing biomedical aspects of their condition(s), with less than half spent considering self-management, personal priorities or daily living [5]. This calls for fundamental redesign of how health services are provided.

Care planning is a systematic approach which can help reshape current routine care and address these issues. Focussed on supporting a more productive conversation between the person and the healthcare professional, it emphasises preparation work by both as an important enabler. The conversation is forward looking, solution focussed, starts with ‘what matters’ to the person [6] and values their role within it. It brings together traditional clinical issues and the person’s lived experience as a ‘meeting between experts’ [7]. It can also provide links to health supporting activities within the wider community [8]. This new way of working involves changes to attitudes, roles, consultation skills, clinic administration and infrastructure being introduced simultaneously. In 2001, care planning was included in the National Service Framework for diabetes [9] building on a growing case for change [10], a developing evidence base [11], experience of clinical teams [12] and national surveys [13]. This emphasised care planning as ‘the process of agreeing a care plan (which) offers people active involvement in deciding, agreeing and owning how their diabetes will be managed’ rather than a plan itself [14].

We have developed, extended and delivered a specific approach to care planning, called the Year of Care approach, which we describe as ‘care and support planning’ (CSP). Starting with diabetes, and learning and adapting as the programme developed, we have transferred the Year of Care approach firstly to other single LTCs and then as a systematic approach to all single or multiple LTCs emphasising ‘people not diseases.’

We worked with UK general practices where there is a systematic approach to the surveillance and management of specified LTCs as part of the Quality and Outcomes (QOF) Framework component of the NHS contract, with recent amendments in Scotland. In many practices most of this work is carried out by practice nurses.

Our approach draws on theoretical models of care, implementation and spread and was developed over time through grassroots work, evidence synthesis, local implementation and evaluation. In this paper, we outline the timeline, methods and settings for this work, highlighting how the ideas and concepts around the delivery of the Year of Care approach to CSP were extended and refined over time. We then describe our model of CSP that emerged from this work, the learning and language that shaped it and key issues for implementation.

## Methods

**Fig. 1:**
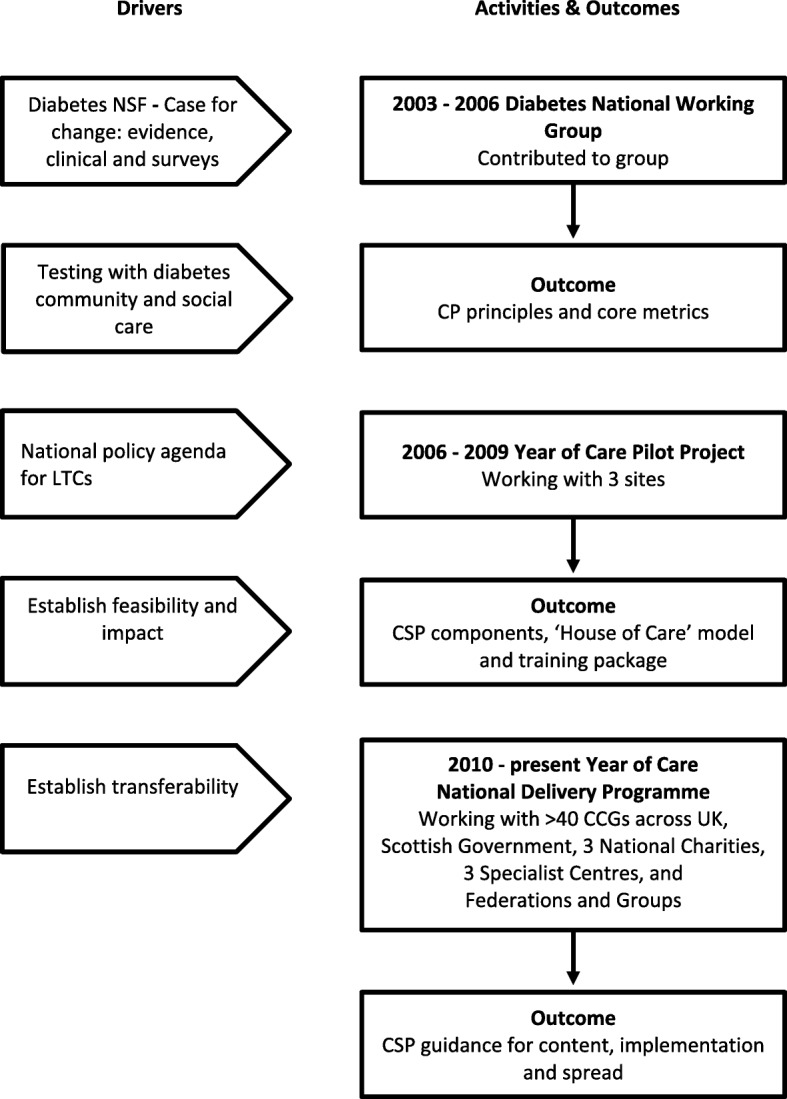
Timeline, drivers, activities and outputs involved in the development and spread of CSP (2003 – present)

For clarity, we describe the process of development of our CSP model (Fig. 1) separately from the process of development of work on implementation and spread. In practice, each strand iteratively informed the development of our work.

### Early activity: 2003–2006

Initially, members of our team (SR, SE) contributed to a national working group [15] which consulted widely in the diabetes community, shared ideas from social care [16], reviewed the theoretical base [17] and reported on what care planning might look like in practice. The developing model emphasised the equal validity of patient and professional ‘stories’ [18] and the importance of social, behavioural, and psychological (notably ‘health beliefs’) components of the consultation as well as biomedical ones. The focus was on identifying patient goals, developing priorities and agreeing actions for the person, the professional and the service, with on-going review.

### Year of care pilot programme: 2006–2009

A national policy initiative to develop personalised and community services [19] provided the funds for a 3-year feasibility study to test these ideas using diabetes as an exemplar for other LTCs. The term ‘Year of Care’ was used to describe ‘all the planned care that a person with diabetes should expect to receive, usually over the course of a year, including support for self-management*’* [20]*.* The role of community activities linked to care planning was emphasised [8]. A steering group including people living with diabetes identified three health communities with diverse demographic characteristics and between 8 and 43 general practices via a competitive process; and funded project managers for 1 year, to support development. A central project manager linked local teams with the steering group and coordinated learning sets. These became key for the project, enabling the sharing of experiences, synthesising ideas and theory [11], as well as articulating and building consensus and establishing training. The early months proved crucial in grappling with a list of 30–40 philosophical and practical issues, which we discovered would need to be changed or addressed differently to enable, what had initially seemed a ‘simple’ idea, to happen in routine general practice. The recognition that these fell into four groups represented in the visual metaphor of the ‘Year of Care House’ (outlined below, see Implementing CSP: working with organisations and teams) and matching the core components of the Chronic Care Model [21]) proved crucial in distilling multiple and complex issues into a delivery programme.

### Year of Care Partnership®: 2010-present

At the end of the pilot the training expertise, learning and resources were brought together as the Year of Care Partnerships (YOCP) team (SE, NL-B, LO, SR) within an NHS Trust with the aim of supporting other health communities to introduce care planning. This small team (less than 5 whole time equivalents) had many years’ experience of clinical care and service design [22], facilitation [23] and patient education [24]. They worked with over 40 health communities [25, 26] and national organisations, across England and Scotland [27] to test the transferability of the principles of care planning to new settings [28, 29] and conditions, including COPD, cardiovascular disease and its prevention [30], musculoskeletal conditions [31], multimorbidity [32], frailty [33, 34] and personal health budgets. This demonstrated the approach was robust, reinforcing knowledge and increasing learning [35].

Initially we engaged new sites via national and local presentations and personal contacts. Subsequently most were recruited by word of mouth from those already involved. A community of practice [36] was established in 2016 to share and explore the developing learning. National workshops and a regular newsletter [37] helped to ensure that new resources were developed collaboratively, building on the experience of local teams. We also worked with national voluntary sector organisations to articulate the components of care planning [38] and demonstrate how these mapped to ‘support planning’ for those using social care [39]. These partnerships influenced the change of language from ‘care planning’ to ‘care and support planning’ (CSP).

### Supporting implementation and spread: 2006-present

Transferring the CSP model to other sites with high fidelity continued in three overlapping phases, with increasing success. During the pilot phase our care planning model, curriculum headlines and list of ‘critical success factors’ were made publicly available for others to use [40]. In the next phase the, now more explicit, ‘Training and Support’ team offered sites a package of support to purchase at cost. Taking a ‘scale and pace’ approach this involved local project managers, building local capacity and cascade training via ‘training the trainers’ [41]. The current approach is focussed on depth and fidelity spreading from local exemplars. The YOCP team works directly with whole practice teams, supporting and challenging them to map pathways, allocate roles and solve issues providing role models and mentorship for local trainers, facilitators and project managers.

### Developing resources: 2006-present

Over 70 resources were developed over the life of the programme (Table 1) with input from both practitioners and patients.

**Table 1:** Resources and their purpose within the programme

Purpose of resource	Examples	How they supported programme delivery
To introduce CSP to people attending the practice	Posters / leaflets / videos for waiting rooms / information for websites individual invitation letters	Each person is prepared for a change in the care process and CSP conversation and their role within it.
To support preparation for each person	Preparation / agenda prompts: a range of material designed to send personal information (test / assessment results) / explanations / and reflective prompts, tailored to different conditions, combinations and generic situations	The person has the same information as the practitioner with time to reflect (with friends and family if desired) prior to the conversation.
To support practitioner preparation	Redesigned assessment tools (e.g. medication, frailty) for self or supported completion by the person	Ensures data collection supports the ethos of ‘working with’ rather than ‘doing to’.
To provide IT components for the 3 clinical record systems used across UK general practice	Predesigned templates for each of 3 systems which enable practices to select patients easily, coordinate appointments, merge test results into letters, record care planning summaries as well as audit and monitor implementation. Read Codes for process components	Enables systems to be set up and ready to go immediately staff have attended training. Reduced administrative workload.
To support quality assurance and monitoring	Practice checklist ‘Quality Mark’	Reflective tools for teams to work together to set up and review how CSP is working in their practice
To support coordinated practice activity / administration	**Practice pack:** All the practice level resources provided in one indexed place for each practice involved	The ‘programme manual’ for delivery teams
To support training	Videos of CSP conversations Slides including a case for change Experiential / interactive activities Case studies / reflective exercises	To support the interactive training programme using a variety of methods and materials.
Overarching Site resources	Case for change Case studies Coordinator guidance Critical success factors A cost modelling tool Sample enhanced schemes Evaluation frameworks	A range of resources to support organisations to develop a local business case; and give an overview of the work involved in setting up and implementing the programme.

These were designed to support activities to deliver CSP such as preparation and local administration, and include self-monitoring tools for teams, training resources and material for wider engagement. They were carefully crafted to ensure that that language and style reflected the ethos of the programme and was consistent across all components. While these could be locally tailored they also act as exemplars and a starting point that ‘made it easy to do the right thing’.

We realised that specific IT components were required to select patients, coordinate appointments, merge test results into letters, and record care planning summaries, as well as audit and monitor implementation. We worked with early adopter practices to develop the necessary functions and codes for each of the three main electronic record systems used in general practice in England and Scotland. These were then offered to other practices. New or modified resources were presented at national YOCP community events and made available to project managers and trainers on a password protected website. This ensured that the programme was refreshed and new content incorporated systematically for quality assurance.

### Developing training: 2006-present

The core training curriculum was designed alongside the CSP model, building on the same theoretical constructs [11], adult learning principles [42], and the educational expertise within the diabetes community and one of the pilot sites [43]. Together with introductory material [44], it was tested in seven further communities, and refined through successive rounds of delivery supported by participant evaluations and trainer reports. Criteria were developed for identifying participants, local support and resource [45]. The curriculum covered the underpinning philosophy of CSP, clinician attitudes and behaviours, organisational issues, CSP consultation skills, and used a goal setting, action planning approach. It provided opportunities to reflect, observe, practise new skills and develop personal and practice-based goals and actions for implementation. Interactive exercises increasingly reflected a multimorbidity approach. Delivered by 2–3 experienced trainers, a doctor was included on the first day to provide credibility and authority to engage GPs.

Initially a GP and nurse from 8 to 10 practices attended for a day and a half separated by around 6 weeks. More recently 4–5 clinical and administrative staff attend from 4 to 5 practices. The half-day has been modified to build on in-practice facilitation that occurs between the training days. As gaps were identified additional modules were designed. This included a one-day version, omitting the focus on set up for staff joining practices where CSP was already in place, and an advanced course for practice nurses based on a training needs analysis. A ‘Train the Trainers’ programme was developed to increase local capacity. Individuals identified against specific criteria attended a hands-on 3-day preparatory course. Each new trainer then delivered the core programme supported initially by an experienced trainer, and then as lead trainer. CSP educational assessors used structured observational tools and a personal development approach when giving feedback, prior to approving most participants to deliver training within their local community.

### Working with theory: 2003-present

A key contribution to the eventual description of the programme was the continuous engagement with theory (Table 2).

**Table 2:** Key bodies of theory and their contribution to the development of the CSP Model, implementation and spread

Theory (Key references)	Key concepts	Role in Development (Validation / Articulation / Design / Transformation)
Empowerment [46], Self-management [47], self-efficacy [48], person centred consultation [49, 50], counselling [51]	Set of theoretical approaches to changing the aims and approaches to diabetes management and the consultation in diabetes and wider.	**Design:** Provided the ‘positive’ case for change; there are things that could work better. Articulated the core conditions for care planning in practice.
Adult education; self-efficacy [52, 42, 48, 53]	Learning is best if grounded in the person’s experience, built up from where people start; supports active learning, recognises importance of building self-efficacy.	**Design:** Informed the consultation model and all training and train the trainers modules.
Chronic care model (CCM) [21]	The 6 components required to work together in the community to enable ‘the engaged empowered patient and the organised proactive system to work in partnership’.	**Validation and articulation:** Components of CSP and The House.
Implementation of evidence-based practice [23, 54]	Identifies 3 components for successful implementation as • Quality of ‘evidence’ • Context for delivery • Method of facilitation	**Design:** Key theoretical driver for YOCP team before and during CSP
Importance and meaning of ‘purpose’ [55, 56]	Importance of: • Being explicit about purpose • Reframing the practitioner role from supporting individual to ‘*manage condition well’* towards ‘*managing life with your condition’*	**Articulation and validation:** Better articulation of purpose of CSP and the language to debate tensions around roles and goal setting with practitioners.
Normalisation Process Theory (NPT) [57–59]	Implementation as work within a social context. Core domains involved in collaboratively implementing complex interventions in complex environments.	**Transformation:** Mapping YOCP success criteria against NPT constructs. Reframing facilitation model, new training exercises, use of NPT tools, redesign NOMAD [60] tool for YOCP.
Importance of context for spread of innovation [61, 62]	Recognising the value of ‘practical wisdom’ to translate core elements of an innovation into a local context to achieve spread.	**Validation, Articulation and Transformation:** Reframing the facilitation challenge as: ‘holding the flame’ vs ‘local tailoring [63]

At key points, often stimulated by the need to articulate the next round of support or respond to challenge and practical learning from sites, theory was sought to help explain and understand findings. This contributed at different points to design, validation, articulation or transformation of elements of the project. It proved invaluable in abstracting principles from task orientated learning, so these could be articulated for the wider community of practice and enable others to tailor their local work while maintaining fidelity to a core set of components.

### Conducting evaluation: 2006-present

From the outset we hypothesised that, through CSP, people will have a better, more useful experience of care, feel more in control of their life with their conditions and have the knowledge, skills and confidence [64] to self-manage more effectively, with longer-term improvements in morbidity, mortality and health service utilization [65]. This was described in a Theory of Change [66] (Additional file 1) and an Outcomes Framework [67]. Two multisite programmes included ‘external’ evaluations [68, 69]. Where local work was commissioned to assess elements of fidelity and impact, this varied in scope, scale and methodology. YOCP activity and learning was recorded throughout in meeting notes, trainer, site and learning event reports.

## Results

**Fig. 2:**
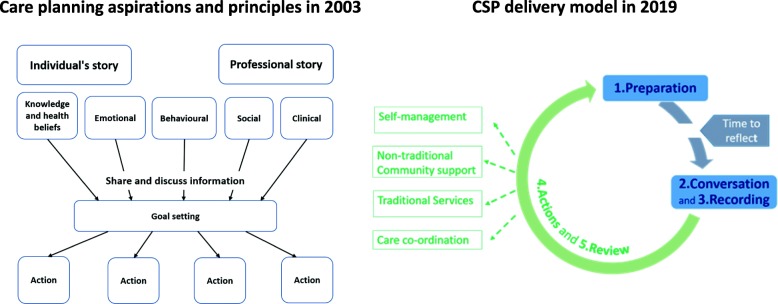
Translating care planning principles into the 5 steps of a generic delivery model of CSP

**Fig. 3:**
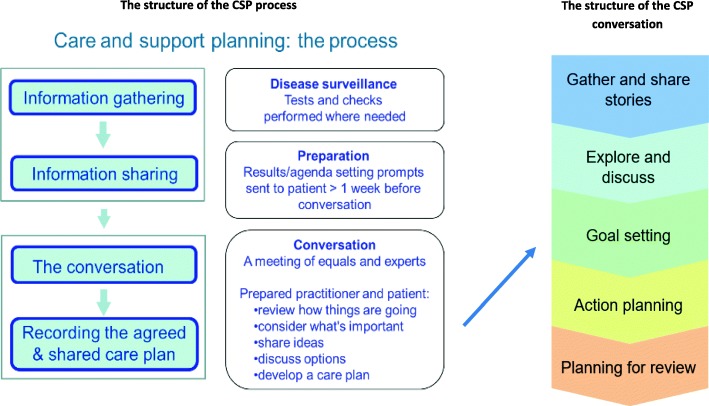
The structure of the CSP process and the CSP ‘conversation’

The aspiration and principles of care planning developed in 2003 were translated into 5 steps – Preparation, Conversation, Recording, Actions and Review – to enable a robust and reproducible delivery model of CSP (Fig. 2). The CSP model is a cyclical process replacing current approaches to planned care. It is expected to occur regularly, usually but not necessarily annually. Space for an uncluttered CSP conversation is achieved by separating out the tasks of disease surveillance and care delivery (e.g. teaching inhaler technique) to be provided as part of the preparation step or in subsequent ad hoc task-oriented appointments. The CSP process is designed to make space for the conversation and enable the person to have the same personal information as the professional with time to prepare before the conversation (Fig. 3). The structure of the conversation was derived from a number of consultation models and reflects common elements rather than favouring one approach [11]. The conversation starts by acknowledging any preparation work and the concerns the person has identified, adds relevant clinical issues if not covered and moves to discuss, debate and prioritise, identify personal goals, develop action plans and review arrangements.

The steps of the model were adapted successfully from diabetes for other single and multiple conditions, prevention and the biopsychosocial complexity associated with ageing and frailty. This offers a generic approach for teams that brings service users and providers together in co-production [70] ‘across a lifetime’ [71] however many issues and conditions may emerge. This approach is also outlined following the TIDieR [72] template (Additional file 2).

### The 5 steps of the CSP model

People in a chosen group (LTC/s, age etc.) are identified from the practice register, usually in their birth month. A small number (current care, malignancy etc.) are excluded in a clinically driven triage process. The majority are invited to take part, with explanatory material on the first occasion.

#### Preparation

The preparation step has two components for most people. The first involves an ‘information gathering appointment’ or sometimes a home visit with a healthcare assistant (HCA) with completion of tests, examinations or assessments needed for condition surveillance. The value of preparing for a new sort of consultation is emphasised with the person. Secondly results are sent to them with simple explanations alongside reflective agenda setting prompts one to 2 weeks before the CSP conversation. Where no direct measurements are involved, the prompts are included with appointment details, and simple information. The practitioner will also prepare by reviewing results, records, and information from colleagues identifying the few issues of real importance for that person from the wealth of clinical information previously collected on condition specific templates.

#### Conversation

The structured conversation with a trained practitioner, usually a nurse or GP, focusses on what is important to each person, bringing together the technical expertise of the professional with the lived experience of the individual, in a solution focused and forward-looking discussion. It starts with concerns the individual has identified, adds relevant clinical issues if not covered, and moves to discuss, debate and prioritise, identify personal goals, develop action plans and review arrangements. Contingency planning, referral for specific clinical or medication review, and links to activities within a supportive community (‘social prescribing’ [73]) may also emerge. Follow up is agreed individually and may not be at routine intervals.

#### Recording

The ‘care plan’ summarises the discussion and is made available for both the person and the system. It includes the issues of importance to the person, ideally in their own words, and can act as an aide memoire for self-management and contingency arrangements (e.g. actions during asthma exacerbations). It may include health professional activities to coordinate ongoing care for those with complex support needs.

#### Actions

The CSP conversation shifts the focus of planned activities away from a medical model towards a social model of ongoing care [8]. Self-management may involve individual actions alone as well as non-traditional formal and informal support from groups and peers. For those with complex needs link workers [74] can build on the issues identified in CSP and support individuals to access an increasing range of community activities. For those who also need specialist, or traditional MDT and social care involvement, CSP initiates an important coordinating function.

#### Review

The review process is determined by the actions agreed during the conversation and may involve self-monitoring of a behaviour change or clinical indicator such as blood pressure, a laboratory check, or a more formal repeat visit to support motivation or to ensure plans are on track.

### Implementing CSP: initial lessons

Ensuring people and practitioners had the same personal information, such as test results, with time and encouragement to reflect prior to the consultation was an early aim of CSP in diabetes. Articulating this as a critical ‘preparation’ step took us longer. Early positive feedback from patients and professionals [11, 20] on a range of benefits was regularly confirmed including by those living with multiple conditions [32, 35]. This included signalling and valuing a new role for the person with time to reflect, leading some to make changes prior to the conversation or raise issues they had not previously thought relevant (such as pain in diabetes). Separating out tests and tasks, done in advance, released time within the consultation for these new topics to be discussed and reduced the need to focus on the computer. Staff concerns that sharing results might increase patient anxiety did not materialise.

Introducing the preparation step can, however, be a significant organisational challenge affecting the whole practice team. It may involve skill mix changes and take weeks to set up. In some practices this concentration of effort led staff to perceive CSP as a system rather than a cultural change and the functional link between a prepared patient and the new style of conversation was sometimes lost. Staff described the activity mechanistically as ‘sending test results’ rather than the enabling function of ‘preparation’. On occasion it was abandoned in the interests of convenience, undermining the integrity of the CSP process. These observations led us to change the language we used in descriptions of the model, training and fidelity metrics. For instance, we relabelled the information sharing leaflets as ‘preparing for your CSP conversation’. As we introduced CSP for people living with increasingly complex issues such as frailty the preparation step also became more complex [34]. HCPs traditionally use specific tools to assess function in these situations. We redesigned these for self-completion by the person in the spirit of ‘working with’ rather than ‘doing to’ and supported practices to identify where best to include these in care pathways.

Over time we learnt that the length of the conversation (20 to 40 min) and the practitioner chosen (usually practice nurse or GP) is influenced by the complexity of issues, prior knowledge of the person, what emerges from information gathering, and the skills and experience across the team. Sites began to build in continuity and patient preferences. We observed that a focus on goal setting sometimes led practitioners to confuse CSP with motivational interviewing [20] unless the role and purpose was explored explicitly. The emphasis on ‘lifestyle change’ in clinical guidelines [75] and perceptions of professional responsibility could create tension for practitioners over the ownership of goals and frustration when traditional ‘clinical goals’ were not met. On occasion staff blamed ‘lack of motivation’, age or social issues when their own priorities were not acted upon. Reframing the purpose of CSP from ‘helping the person to manage their condition’ towards ‘helping the person to manage their life with their condition’ [55, 76] - which might include specific condition management tasks - helped practitioners to value a wider range of components of the conversation. These included building a therapeutic relationship, an empathic approach [77], and recognising the person’s problem-solving abilities; as well as striving to increase the person’s specific knowledge, skills and confidence [64]. This was particularly relevant to those with multimorbidity, but it was a core issue for everyone.

### Implementing CSP: working with organisations and teams

**Fig. 4:**
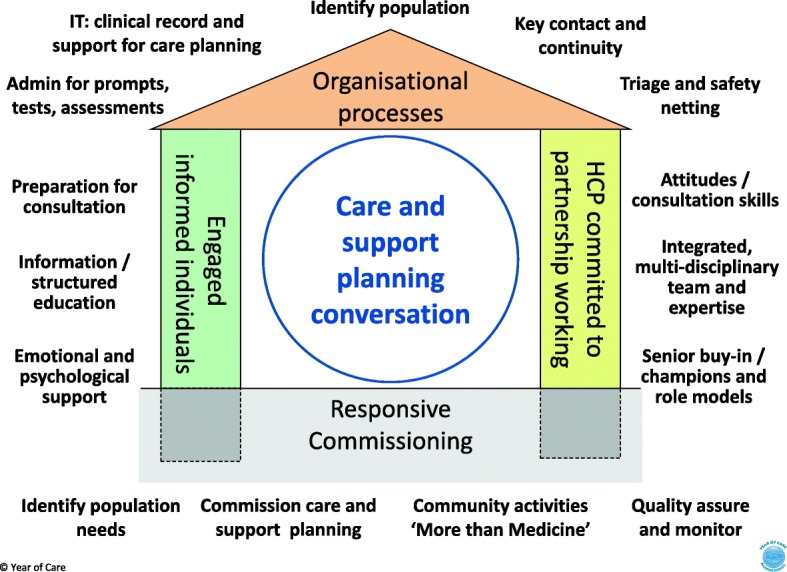
The Year of Care House (‘The House’)

We devised the Year of Care House (‘The House’) (Fig. 4) as a checklist of essential enablers of CSP, and a metaphor emphasising that the walls, roof and floor must be addressed together. YOC pilot sites assigned all the issues they identified to four groups which became the walls, roof and foundations of the Year of Care House. This emphasises that effective CSP consultations rely on these elements working together in the local healthcare system: an engaged, empowered person working with health care professionals (HCPs) committed to a partnership approach (the walls), supported by appropriate/robust organisational systems (the roof) and underpinned by responsive whole system commissioning. In Scotland the foundations of The House were adapted to reflect the different organisational arrangements within the Scottish NHS. The House acts as a check list for what needs to be in place; is a metaphor for the interdependence of each part (if one is weak or missing the structure is not fit for purpose); and provides a flexible framework to enable communities to get started and design the sort of House that suits their population. When used in the training programme, a blank outline of The House is provided, and participants are asked to consider what needs to be in place to deliver and support the CSP process and then reflect on current local services. The concept of the Year of Care House proved useful in helping local groups focus their support programmes and was taken up by others as a way to organise person centred programmes of work [26, 78–80]. The components shown in Fig. 4 are those that have been consistently identified by teams across multiple diverse health communities [78].

Many of the elements that make up The House were common across sites. These included the need for IT support to get the letters and codes embedded in local systems, senior clinical leadership to secure changes in practice organisation and oversight groups which were visibly led and welcomed feedback. Other components often benefited from local design, and this led to an increasing repository of adaptations to be shared across the CSP community. Exploring the use of language proved critical. For example, staff may assume that ‘care plans’ and ‘treatment plans’ have the same purpose, and that templates are lists of traditional ‘clinical’ issues to be ticked off during the conversation. The term ‘care and support planning’ is often not recognised even by ‘patients’ who have participated in CSP and value it. Local terms (e.g. ‘yellow letter’, ‘MOT’) need to be agreed and used across the team. Partnerships with voluntary organisations and patients influenced the language we used, replacing ‘consultation’ with ‘conversation’. Where confusion between ‘end of life planning’ and ‘care planning’ was identified, local documentation was changed to ‘health planning’.

A focus on the left wall (‘engaged informed individuals’) led sites to explore local demographic, cultural and health literacy issues. Some vulnerable communities [20] needed additional resources to engage but once involved they participated fully [81]. Examples included practice and faith based introductory sessions, links with education programmes, preparatory material in a variety of formats, including colour coding, home visits and greater use of the telephone. Although many workforce components of the right wall (‘health care professionals committed to partnership working’) were common, these needed tailoring to practice skills and demographics. Expanding the role and number of administrative and non-qualified staff made more time for nurses during the conversation. Working at the top of their grade, HCAs could also reduce non-attenders by engaging patients at the outset of the process, leading to their greater recognition within the team, job satisfaction and career development [82].

Within the CSP conversation attending to the ‘person’ not the ‘condition’, meant practitioners needed to be ‘expert generalists’ [83] handling a range of health, social and behavioural issues, during one discussion. We observed that this was easier for GPs than practice nurses, whose role and expertise in the UK has been incentivised towards single disease surveillance using a template driven approach (QOF) [84]. GPs could also draw on core communication skills, but this is not part of basic training for practice nurses, to whom much of LTC management has been delegated. Master classes in conditions that nurses were unfamiliar with and further training in handling difficult conversations proved useful. The most successful practices used in-house support and mentorship to smooth the transition to new roles and ways of working.

### Implementing CSP: enabling scale and spread

The core challenge driving the changes in our developing approach to spread was how to maintain fidelity to the CSP model during transfer to other communities, while building on local ‘practical wisdom’ [85] to secure ownership and success. Our learning over the whole programme was that this tension could be used constructively if explicitly recognised, and our approach, resources, training and support were adapted to manage this positively.

In parallel with the pilot phase a few independent sites resourced local programmes to introduce CSP (one across a whole regional economy), using early published descriptions of the model [40] as part of local strategic plans. They had limited contact with the YOC team, training was not yet available, and they stressed ‘don’t tell us what to do’*.* Using didactic approaches, they found that CSP was difficult to introduce, with poor clinical engagement or culture change; none were sustained. As the core training programme and train the trainers became available, we developed productive relationships with local managers who requested ‘please tell us how to start’ and effective CSP began to be established. Apart from training days we had no direct contact with practice delivery teams. Two members from each practice were expected to attend, return to the practice following training, convince colleagues to embrace the approach and make personal and practical changes. Although it was recommended that attendees should be ‘people with power to make change’ this proved too big a challenge for some.

Local managers also struggled to use the ‘critical success factors’ identified in the pilot programme to support local practices after training. We turned to theory to reframe issues and this became a turning point both for local managers and our own team. Normalisation Process Theory (NPT) [86] questions assumptions about the delivery of complex interventions in a complex environment, using a sociological perspective to challenge the over-reliance on psychological theories of behaviour change in health settings [53, 87] which had dominated our thinking. With support of the NPT innovators, we reframed the task orientated list of ‘critical success factors’ under the four theoretical concepts of NPT. Using NPT tools [58, 59] in workshops enabled project managers to recognise barriers based on human relationships and team interactions, and to design local solutions. We promoted ‘coherence’ as an essential principle for action rather than the aspiration of ‘consistent vision across the organisation’*.* This engaged steering groups and practice teams with the importance of fidelity. A redesigned training exercise focussed participants on what was different about the new way of working enabling clinicians to discard the belief that ‘we do this already’ and stimulated discussion and greater consensus about ‘purpose’.

Despite these improvements this cascade model of support was slow, exacerbated by frequent changes in local staff, short term funding and a prevailing emphasis on ‘pace and scale’ to be achieved predominantly via training. However, we observed that where individual practices embraced CSP for the majority of their LTC patients and the whole practice was involved, they acted as exemplars, hosting visits from other communities and proved a powerful driver for adoption both locally and beyond [82]. We changed the way we worked to capitalise on this, working directly with ‘early adopter’ practices identified at ‘taster sessions’, using our experienced facilitators to support them to map processes, discuss roles and challenge individual issues [57] either immediately before or after they attended core training. The positive experience this generated spread by word of mouth and helped to recruit subsequent cohorts.

Local capacity building needed to embrace this facilitation approach [23]. This required an acknowledgement of the importance of context [85] and the personal skills and attributes involved in ‘holding the flame for CSP’ while ‘supporting local tailoring’. Helping steering groups to distribute the roles of project manager, trainer and practice facilitator across a local long-term support programme became an early action at new sites. We developed a 3 day ‘facilitator programme’ and direct work with practices provided opportunities to offer a ‘mentorship’ model for local support staff. YOCP facilitators demonstrated how to use observed behaviours and language to identify cultural barriers at team level and how to share practical solutions to common problems collated from the wider community of practice. Debriefing immediately after practice sessions and support by phone as issues emerged helped develop local staff. This current approach to implementation is outlined following the TIDieR template [72] (Additional file 3).

### Impact of CSP

The achievement of improved patient experience, practitioner job satisfaction, health behaviours and outcomes, team work, practice organisation and resource use reported in the pilot [20] have been repeated throughout the programme [88]. CSP is now ‘business as usual’ in a majority of practices across several CCGs [32, 88]. The demonstration of greater savings for a practice as patient complexity increases [89, 90] has helped to engage new teams. In diabetes, improved biomedical outcomes take time to emerge across populations [20] but have been sustained [91]. Large managed programmes have seen stepwise improvement in completion of the core processes of diabetes care [88] moving from within the bottom 10% to first place [91] and from 119th to 5th place [92] in national comparisons [93].

## Discussion

In this paper we describe the development, delivery and spread of CSP for people living with LTCs, that addresses the challenges to current provision. Our CSP model recognises the person as the main agent in living with and managing their life with these conditions based on their values and capabilities. Consequently, the focus of our design has been to support them to do this, to value their role and seek to maximise engagement, involvement and capacity for self-management linked with appropriate bio-medicine.

The CSP approach turns the components and key relationships described within the Chronic Care Model (CCM) [21] into a set of practical steps, bringing together traditional clinical expertise and lived experience in a systematic process of co-production [70]. It adds to the body of international work based on the CCM [94] by demonstrating implementation, transferability and sustainability of CSP practice within the UK. But it differs by describing the delivery principles and core components, in a process of reverse engineering, from grass roots experience. This strengthens the core message of the models, aiding transferability and adaptability to the changing needs of patients and local communities.

The process of developing the five steps of CSP and four elements of The House has brought two components into sharper relief. *A planned preparation step* emerged as a key enabler of a ‘better conversation’ as well as supporting engagement beyond the consultation. Further focused research to understand this and the mechanisms involved, in greater depth, could enhance delivery of the process. *The mutual dependence of all the elements* emphasised by the metaphor of The House suggests that any phased implementation of CSP should be based on small scale adoption of the whole model, rather than wider introduction of a few elements; with the temptation to implement systems before ethos being a particularly damaging example. Circumscribed interventions to support people with LTCs [65] such as improved consultation skills, behaviour change, education or group programmes might have wider reach and sustainability within such a whole system approach.

The House framework supports both local implementation and spread of CSP within a single image. As an enabler of local implementation, the essential philosophy and systems it depicts are almost unchanged over 10 years. Future descriptions, evaluations or comparisons of CSP need to demonstrate fidelity to these aspects [72] to be meaningful, or to evidence the impacts (on patient and service delivery outcomes) of variations from this model. As a framework to support spread The House offers an easily assimilable description of the ‘non-negotiable’ elements, with the implicit invitation to the new ‘owner’ to design the details creatively to match the demographics, geography and customs of their local population. However, in our experience description of the components alone or a self-contained training programme, however well designed, is insufficient to enable new ‘House builders’ to achieve a sound structure without a facilitation process alongside.

Clarity around the core concepts of CSP and the components of the walls and roof of the House has enabled us to support reliable delivery outside the UK where the contextual elements reflected in the floor, including population characteristics, funding and organisational arrangements may be very different. Our successful joint work with teams in Singapore and Jersey where there are private and insurance based health systems illustrates some of the practical issues which may be of interest to others who wish to introduce this way of working.

The key drivers in each case were the strategic fit with local aspirations for greater involvement of the public in their own health and healthcare and local leaders who had serendipitously engaged with the programme experientially and were personally confident in the approach. Via direct contact with the YOCP team we were able to transfer the philosophy, ideas and expertise to initiate and build local capacity. The issues of large numbers of people with diabetes and the temptation to modify core elements of the model to fit traditional local systems, roles, mindsets, specialist sites of care and incentives had to be worked through. Arrangements for each element of the programme including preparation, the new style of conversation and commitment to support for self-management were challenged and addressed to maintain fidelity to the core principles. This resulted both in effective new pathways and local leaders and facilitators who understood the new way of working and were committed and supported to taking forward high-quality implementation and spread. Local health communities provided funds to support this transition work, free up local teams to take part and support long-term maintenance and quality assurance.

### Strengths

The strengths of our approach include its theoretical foundation [11, 17, 95, 96] and sustained period of grass roots testing in environments where it is to be used routinely. It is widely applicable to single or multiple conditions and increasing complexity. It can bring together physical and emotional health and wellbeing as part of planned care ‘across a lifetime’, replacing current fragmented approaches for general practice teams. It offers commissioners a common approach to issues previously specified separately with economies of scale, training and project management and links between traditional clinical care and community activities.

At a time when general practice is under exceptional pressure in the UK, practitioners have reported CSP as ‘a better way to work’, sparking enthusiasm in some, valuing relationships over check lists and providing a lever for better teamwork and role definition. A range of intangible improvements to motivation need formal testing but seem to be driving peer to peer uptake [32]. Although informal comparisons of like for like resource use across a practice suggest that introducing CSP is either cost neutral or cost saving especially for those with multiple conditions, no economic evaluation has been undertaken. Anecdotal reports of subtle changes in other patient and practitioner behaviours across participating practices suggest that much may be gained from an in-depth study of ‘what happens in a CSP process?’ using ethnographic approaches that can inform a detailed economic analysis of its impacts.

CSP is an important component of the drive for personalised care within health policy [97] supporting the need for fundamental change to deliver a high value health service for people living with LTCs [98, 99]; and this paper demonstrates how this can be achieved. It advocates driving change from a focus on the person’s experience and relationships with practitioners, instead of large scale organisational and financial change. Rather than targeting groups of people with LTCs for potential interventions [97], CSP adopts a universal approach [100] in which every person has an opportunity to share in defining their own need, so resources can be provided proportionately. Tools and checklists to assess fidelity to the CSP model as part of core training enable exemplar practices to act as laboratories for further study, observation and teaching, and provide a common language and starting point for comparisons with similar approaches in the voluntary and social care sectors.

### Limitations

The work we report is a longitudinal feasibility study of theory informed service development. Core components were developed early and then subjected to scrutiny across multiple sites, but have not been tested formally against comparators or usual practice, and this may not be helpful when assessing such complex change [101]. Improvements in behaviours and activities are self-reported, and positive change in diabetes indicators across populations have often been part of wider system changes so attribution specifically to CSP is not possible from data collected alongside service implementation, as reported here. Emerging issues have been addressed throughout the implementation process, but the possibility of unrecognised negative consequences either within practices or beyond cannot be excluded.

The extra resource needed to introduce and embed CSP including time, effort, training and facilitation must be set against the benefits which can currently only be directly linked to reported improved patient and staff experience. For some teams and practitioners, the balance favours change, but for commissioners and provider groups who fund implementation activities this may not be the case. The financial benefits of improved engagement in health and self-management [65] take time to emerge and may accrue to others. Introducing CSP as a new way to work rather than an ‘add on’ project, and the multiple components introduced simultaneously, has meant that quality and speed of implementation has varied across practices. New attitudes and habits need reinforcement so methods to identify and ensure fidelity to the key elements are important. This is easier to establish for processes than for attitude change. Getting inside the consultation to observe the behaviours and content which are at the core of the rationale for CSP has proved hardest.

Although we have collated metrics to assess various aspects of CSP and its impact, none have been specifically designed for CSP. A culture of poor use of data for improvement [102, 103] at team level, where benefits are judged by personal experience of day to day practice and are in tension with external performance management exacerbates this issue. This has limited the data we have been able to collect in the context of service development, rather than formal scientific evaluation. This could be addressed and encourage reflexive practice via resourcing time for practices to identify and monitor key outputs and outcomes of their work [104] and the development of metrics specifically designed to support this.

## Conclusions

In this paper we have outlined how we developed our specific care and support planning programme and concomitant implementation and spread strategy over 15 years, incorporating a process of grassroots work, evidence synthesis, local implementation and evaluation. Starting with diabetes, and learning and adapting as the programme developed, we have transferred the approach firstly to other single system long term conditions and then to a systematic approach to all single or multiple long-term conditions with an emphasis on ‘people not diseases.’ It enhances the opportunities within routine planned contact with health professionals and underpins a person-centred approach. It explicitly links biomedical and psychosocial care to non-traditional community resources that support wellbeing.

We have clarified the process, content, purpose and benefits of CSP within the very complex and constantly changing setting of primary care. This requires constant attention to detail and context, high levels of fidelity to the principles of CSP, and an ability to apply the principles flexibly in different macro and micro-environments. It is supported by a training programme for local teams to get started, and close facilitatory relationships with delivery teams providing specific content knowledge, skill set and credibility over and above training to secure local ownership.

CSP has the capacity to be transformative both for people with LTCs and for health professionals and recaptures a relational approach to care, with transactional elements in the background. It offers the opportunity for healthcare more widely to be transformed for people with LTCs. Options for applying this learning at scale within an effective support and evaluation programme now need to be explored.

## Supplementary information

**Additional file 1.** A theory of change for CSP.

**Additional file 2.** TIDieR framework [72] describing key components of CSP and its implementation using the YOC approach.

**Additional file 3.** TIDieR framework [72] describing YOC approach to implementation and key components of successful spread of CSP.

## Data Availability

There was no specific data collected as part of this research.

## Abbreviations

‘The House’: Year of Care House
CCG: Clinical Commissioning Group
CCM: Chronic Care Model
CSP: Care and support planning
HCA: Healthcare assistant
HCPs: Healthcare professionals
LTCs: Long term conditions
MDT: Multi-Disciplinary Team
NHS: National Health Service
NPT: Normalisation Process Theory
NSF: National Service Framework
QOF: Quality and Outcomes Framework
YOCP: Year of Care Partnerships
