# Supplementary material for: The Year of Care approach: developing a model and delivery programme for care and support planning in long term conditions within general practice
Source: BMC Fam Pract. 2019 Nov 8;20:153. doi: 10.1186/s12875-019-1042-4 (PMC6839214; doi:10.1186/s12875-019-1042-4)
Supplement: Supplementary file 1 — Additional file 1. A theory of change for CSP. [file 12875_2019_1042_MOESM1_ESM.docx]

additional file 1: A theory of change for CSP

**5-step process**

Involving changes to:

- Attitudes
- Skills
- Clinic infrastructure

**People and professionals are PREPARED**

**by:**

- Sharing collected information
- Prompts
- Time for reflection

(separating tasks from conversation)

**‘Better conversation’**

Bringing together:

- Expertise
- Patient’s and professional’s agenda
- Forward looking
- Solution focussed

**Improved:**

- Patient experience
- Knowledge, skills and confidence to self-manage outside clinic setting
- Staff job satisfaction
- Use of clinic resources
- Opportunity to address inequalities
- Use of community resources
- Coordination of care

**Outcome:**

- People have greater sense of control/well-being/agency
- Improved clinical/lifestyle issues
- Improved staff motivation
- Better use of system wide resources
