# Supplementary material for: The Year of Care approach: developing a model and delivery programme for care and support planning in long term conditions within general practice
Source: BMC Fam Pract. 2019 Nov 8;20:153. doi: 10.1186/s12875-019-1042-4 (PMC6839214; doi:10.1186/s12875-019-1042-4)
Supplement: Supplementary file 2 — Additional file 2. TIDieR framework [72] describing key components of CSP and its implementation using the YOC approach. [file 12875_2019_1042_MOESM2_ESM.docx]

##### additional file 2: TIDIER FRAMEWORK [73] DESCRIBING KEY COMPONENTS OF CSP AND ITS IMPLEMENTATION USING THE YOC APPROACH

| **TIDieR Heading** | **Core component** |
| --- | --- |
| Brief Name | Care and support planning (CSP) model and delivery - using Year of Care approach |
| Why | Current model of routine care for people living with LTCs does not support them to manage their lives with their conditions, support self-management, coordinate care or link with community resources |
| What | CSP is an ethos driven, systematic 5-step approach to planned care for people living with (or at risk of) long term conditions which ensures people (and practitioners) are prepared for a new style of conversation with a trained healthcare practitioner which focusses on ‘what matters to each person’, brings together professional expertise and lived experience, is forward looking and solution focussed, usually results in actions for the person and /or professional/service and is summarised as a ‘care plan’, with individualised review.  It replaces current planned care. Usually annual cycle - not a one-off review. |
| Who provides | A multidisciplinary general practice team / or other team with primary responsibility for all aspects of care for an individual within a local pathway. Clearly identified roles and relevant training including expanded admin team, health Care Assistant (HCA) for information gathering appointment, GP / senior nurse for consultation, other expertise for support e.g. pharmacy /mental health etc. May be linked to community activities via a link worker based in general practice or community. |
| How | A single process however many conditions / issues are involved. Recall from practice register in birth month; triage of those unsuitable; attendance at an information gathering appointment / or home visit if there are tests /tasks to be performed; reflective prompts sent to person with results of tests and tasks and explanatory information 1-2 weeks before a consultation appointment with trained/ supported professional. A structured conversation usually based round goals and action plans is summarised and recorded for the person and the system, includes individualised review and may include contingency plans, referrals and community links. |
| Where | General practice (or different team base) |
| When / how much | Usually annually in on going cycles, with potential for individualised review in between. Later cycles may be at different agreed /individualised intervals. All steps required for all people. Time allocation for information gathering (usually 10 -30 minutes), and for CSP conversation (usually 20-40 minutes) determined by local decisions and patient complexity. Resource neutral or saving if multimorbidity across a practice. |
| Tailoring | The structured conversation is tailored to each individual by design. Other steps including preparation (content of shared information) and administration (media design /site e.g. use of phone, IT links /home visits etc.) are tailored to groups of people with common features /requirements e.g. demographics, conditions or levels of complexity |
| Modifications | Provided core philosophy and inclusion of all steps are adhered to almost every aspect of delivery is modifiable by local teams, to account for local demographics, health literacy, skill mix, branding, preference and good ideas. |
| How well (planned) | Resources including exemplars, pathway design, training, structured support, fidelity and quality markers available |
| How well (actual) | See TIDieR for implementation and spread |
