# Supplementary material for: The Year of Care approach: developing a model and delivery programme for care and support planning in long term conditions within general practice
Source: BMC Fam Pract. 2019 Nov 8;20:153. doi: 10.1186/s12875-019-1042-4 (PMC6839214; doi:10.1186/s12875-019-1042-4)
Supplement: Supplementary file 3 — Additional file 3. TIDieR framework [72] describing YOC approach to implementation and key components of successful spread of CSP. [file 12875_2019_1042_MOESM3_ESM.docx]

##### ADDITIONAL FILE 3: TIDier framework [73] DESCRIBING YOC approach to implementation AND key components of successful spread of CSP

| **TIDieR Heading** | **Core component** |
| --- | --- |
| Brief Name | Implementation and spread of CSP using the Year of Care approach |
| Why | CSP is a complex intervention with multiple steps and components to be introduced into complex health care settings involving changes to attitudes, skills and infrastructure. It requires active management to maintain fidelity during local tailoring process. |
| What - resources | A team with expertise, experience and understanding of philosophy, general practice culture and processes, training delivery and assessment, resource design and production, pathways and service design and team facilitation; working with local oversight team to include leadership, resource acquisition, project management, training and long-term practice support /facilitation, quality assurance, monitoring linked with delivery of supportive services in community. |
| What- procedures | Using Year of Care House as delivery framework to deliver all 5 steps of CSP (See TIDieR plan 1). Addressing engaged patient (left wall), HCP committed to partnership work (right wall) organisational process (roof), and resource use, community activities and quality assurance (floor). |
| Who provides | Delivery team level: In practice operational /oversight group. Support for practice level delivery teams: local leadership/ steering group supporting and resourcing practice delivery via close working with YOCP team on engagement activities, training, support to practices including IT and practice facilitation. Support for leadership/ steering group in new site: YOCP team or others by agreement - trained, mentored and supported by them. |
| How | Developing practice exemplars: Early adopters identified at taster events, attend training linked with practice visits in groups of 4-5 practices. YOCP works directly into practices, to map pathways, discuss roles and skill mix, identify issues and support practices and link with oversight team to resolve these. Building local support capacity: Works with local leadership teams/ project managers to plan spread across community, including role modelling /mentoring and specific training trainers and facilitators modules. |
| Where | Within local GP practices /local training venues. Training trainers at multisite venues. |
| When / how much | First 6-12 months – with gradual transfer of skills and experience – on going contact via community of practice and updated resources |
| Tailoring | Balance of support between YOCP and local sites dependant on local skills and experience - negotiated |
| Modifications | Provided the core model of CSP and all elements of The House are addressed at practice level, using fidelity tools and quality markers the balance of project management, training and facilitation can vary depending on local skills and workforce. |
| How well (planned) | Suitable for all teams providing routine and preventative services for people living with single LTCs or multimorbidity, increasing complexity, ageing and frailty and preparation for end of life. |
| How well (actual) | Single and multiply practice sites across England / Scotland carrying out CSP using YOCP approach for people living with single and increasingly multiple conditions; increasing percentages of practices in a health community population (e.g. 59/64) - increasing number of conditions involved and increasing % of practice population e.g. up to 20% practice population. |
